# Adaptation of the human aryl hydrocarbon receptor to sense microbiota-derived indoles

**DOI:** 10.1038/srep12689

**Published:** 2015-08-03

**Authors:** Troy D. Hubbard, Iain A. Murray, William H. Bisson, Tejas S. Lahoti, Krishne Gowda, Shantu G. Amin, Andrew D. Patterson, Gary H. Perdew

**Affiliations:** 1 Graduate Program in Biochemistry, Microbiology, and Molecular Biology; 2 Department of Veterinary and Biomedical Sciences, The Pennsylvania State University, University Park, PA 16802; 3 Department of Environmental and Molecular Toxicology, Oregon State University, Corvallis, OR 97331; 4 Department of Pharmacology, Penn State College of Medicine, Hershey, PA 17033, USA

## Abstract

Ligand activation of the aryl hydrocarbon (AHR) has profound effects upon the immunological status of the gastrointestinal tract, establishing and maintaining signaling networks, which facilitate host-microbe homeostasis at the mucosal interface. However, the identity of the ligand(s) responsible for such AHR-mediated activation within the gut remains to be firmly established. Here, we combine *in vitro* ligand binding, quantitative gene expression, protein-DNA interaction and ligand structure activity analyses together with in silico modeling of the AHR ligand binding domain to identify indole, a microbial tryptophan metabolite, as a human-AHR selective agonist. Human AHR, acting as a host indole receptor may exhibit a unique bimolecular (2:1) binding stoichiometry not observed with typical AHR ligands. Such bimolecular indole-mediated activation of the human AHR within the gastrointestinal tract may provide a foundation for inter-kingdom signaling between the enteric microflora and the immune system to promote commensalism within the gut.

The aromatic bicyclic indole composed of benzene fused to a pyrrole ring is found abundantly in nature as a metabolic product and as an indolyl moiety component of numerous biological molecules utilized by all microorganisms, plants, and animals. Indole is the functional group that defines the amino acid tryptophan and is a chemical component of the neurotransmitter 5-hydroxytryptamine, the hormone melatonin, and the plant signaling and pigment molecules auxin and indigo, respectively. In bacteria, indole and indolyl compounds, including isatin and various hydroxy-indole derivatives, function as intra- and inter-species signaling molecules across bacterial populations, where they are involved in biofilm formation, bacterial motility, plasmid stability, virulence and antibiotic resistance^1^,^2^,^3^,^4^. Bacterial synthesis of indole was first recognized in the late 1800’s and is the result of *tryptophanse* (TnaA) dependent metabolism of tryptophan^5^,^6^,^7^. The gastrointestinal tract, which may contain >10^12^ enteric bacteria, harbors numerous species (*e.g. E. coli*) with the capacity to synthesize indole; consequently indole is present at high micromolar concentrations within the intestinal lumen and feces^3^,^8^. Recent evidence has suggested that bacterial-derived indole also provides a basis for signaling between intestinal bacteria and the host, resulting in modulation of epithelial gene expression and the maintenance of epithelial barrier integrity^9^,^10^,^11^. The mechanism(s) whereby intestinal epithelial cells sense and respond in a targeted fashion to bacterially generated indole have yet to be elucidated. However, previous reports have highlighted the capacity of the aryl hydrocarbon receptor (AHR) to respond to a number of indolyl metabolites, including indoxyl-3-sulfate, 6-formylindolo[3,2*b*]carbazole (FICZ), kynurenine, kynurenic acid, tryptamine, and indole-3-acetate (Fig. S1), thus positioning the AHR as a candidate indole receptor^12^,^13^,^14^,^15^,^16^,^17^. Furthermore, recent studies have demonstrated that the AHR participates in the establishment/maintenance of intestinal homeostasis, which includes epithelial barrier integrity, regulation of commensal bacterial phyla, and protection from pathogenic insults^18^,^19^,^20^,^21^,^22^,^23^. The protective action of the AHR is dependent upon ligand-mediated activation with the diet, providing a source of presumptive ligands^18^. The complimentary observations that the AHR is required for optimal gastrointestinal health, indolyl compounds represent an expanding class of AHR ligands, and that enteric bacteria can generate such compounds *in situ* has prompted us to examine whether indole is a ligand for the AHR.

Our findings presented here demonstrate that indole and 3-methyl indole exhibit species-specific AHR agonist activity, activating human but only marginally activating the mouse AHR. *In silico* modeling data suggests that such species specificity may be a consequence of a bimolecular (2:1) stoichiometry between indole and the ligand-binding domain of human AHR. These data suggest that activation by indole may establish the AHR as a host sensor of the enteric bacterial population through their *TnaA*-dependent metabolism of tryptophan and provide an additional link between the diet, gut microbiota, AHR, and gastrointestinal homeostasis.

## Results

### Human AHR is permissive for indole-mediated activation

**Figure 1:**
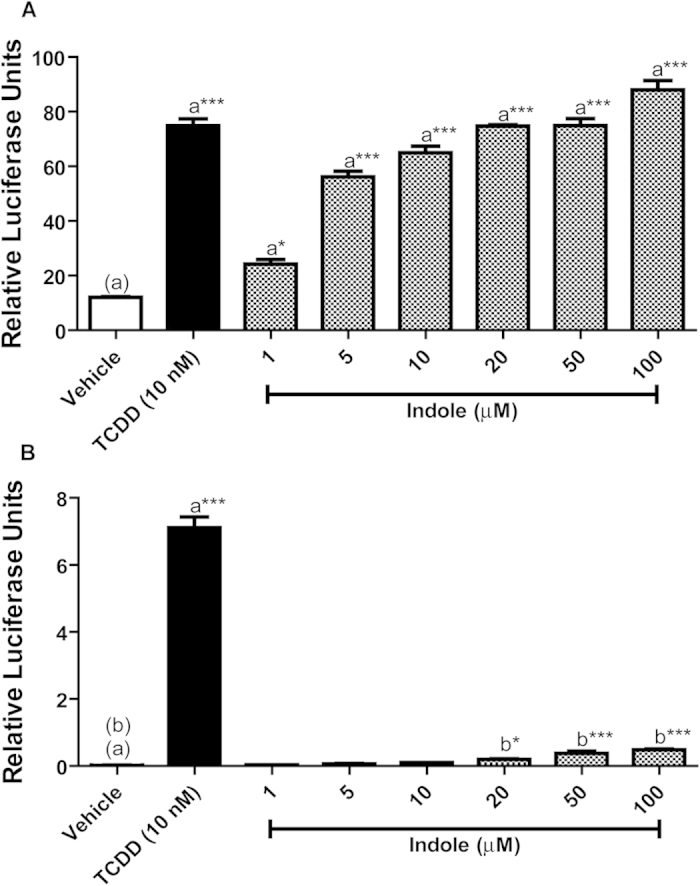
Indole dose-response assessment of AHR-dependent activity. (**A**) HepG2 (40/6) cells and (**B**) Hepa 1.1 cells were treated as indicated for 4 h; cells were lysed, and luciferase activity was measured.

To investigate the impact of indole upon AHR-mediated gene expression, human HepG2 (40/6) cells stably harboring an AHR responsive luciferase reporter construct were incubated with vehicle, 10 nM TCDD or increasing concentrations (1–100 μM) of re-crystallized indole as indicated (Fig. 1A). Exposure to indole resulted in a dose-dependent increase in reporter expression with an EC_50_ ~ 3 μM. A significant 2-fold induction over vehicle treated was observed at 1 μM and maximal 7-fold expression evident at 100 μM, the highest concentration examined and equivalent to the induction obtained with a saturating dose of the prototypical AHR agonist, TCDD. Such data indicates that indole stimulates canonical dioxin response element (DRE)-dependent AHR-mediated gene expression in the context of human AHR.

The AHR is known to exhibit species-dependent sensitivity with regard to its activation potential. We therefore examined the capacity of indole to influence AHR-mediated gene expression in mouse Hepa1.1 cells stably harboring an AHR responsive luciferase reporter construct. Hepa1.1 cells were incubated with vehicle, 10 nM TCDD or increasing concentrations (1–100 μM) of indole as indicated (Fig. 1B). In contrast to the human HepG2 (40/6) cell line, exposure to indole resulted in a modest but significant increase in reporter activity at 100 μM. This represented only 7% of the activity exhibited with 10 nM TCDD, thus suggesting that indole is a weak partial agonist for the mouse AHR. Further examination of the sensitivity of AHR-mediated gene expression by indole was performed using the rat H4IIE1.1 luciferase reporter cell line and revealed that rat AHR-mediated gene expression, similar to mouse is weakly responsive to induction by indole at the doses examined (Fig. S2). Contrary to these data, previous studies have reported that indole is antagonistic with regard to AHR-dependent gene expression^17^. In order to validate the observed inductive capacity of indole and eliminate potential AHR agonist contamination of our indole source, we re-examined the sensitivity of human HepG2 (40/6) using re-crystallized, HPLC-purified and commercial grade ^1^H-NMR validated indole. AHR-mediated reporter expression in HepG2 (40/6) cells exposed to 10 μM recrystallized or HPLC-purified indole yielded essentially identical activity. In contrast, commercial grade indole failed to exhibit significant luciferase reporter activity (Fig. S3). Perhaps explaining the differing results obtained by others^17^. These data indicate that human AHR, in contrast to rodent, is sensitive to indole-mediated activation at low μM concentrations.

### Indole stimulates human AHR-mediated target gene expression

**Figure 2:**
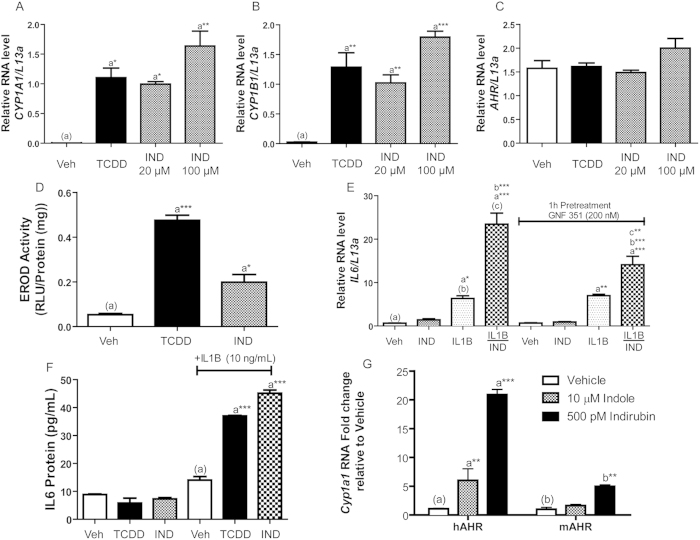
Indole stimulates AHR-target gene expression. (**A**) Expression of AHR-responsive *CYP1A1*, (**B**) *CYP1B1*, and (**C**) *AHR* within Caco2 cells was determined through qPCR analysis following 4 h of treatment with vehicle, TCDD (10 nM), or indole (IND) at the indicated dose. (**D**) The mean CYP1A1 enzymatic activity was measured in Caco2 cells following 12 h treatment with DMSO, TCDD (10 nM), or Indole (100 μM) and 3 h incubation with luciferin-CEE reagent. (**E**) *IL6* expression within Caco2 cells was determined by qPCR following 4 h treatment with indole (20 μM) with or without the addition of IL1B (10 ng/mL), AHR dependence was evaluated by 1 h antagonist pretreatment using GNF 351 (200 nM). (**F**) IL6 secretion by Caco2 cells was determined by ELISA following 24 h treatment with vehicle, TCDD (10 nM) or Indole (100 μM), with or without the addition of IL1B (10 ng/mL). (**G**) *Cyp1a1* gene expression within isolated peritoneal macrophages from C57BL6 and AHR humanized mice were evaluated by qPCR following indicated treatment of 4 h.

To examine indole-mediated human AHR activation within the context of endogenous gene expression rather than a heterologous reporter system, quantitative AHR target gene expression was assessed in colonic epithelial Caco2 cells exposed to vehicle, 10 nM TCDD, 20 and 100 μM indole, as indicated (Fig. 2). Induction of the AHR target genes *CYP1A1* and *CYP1B1* by TCDD and indole was consistent with the previous reporter cell line data with 20 μM indole displaying inductive capacity equivalent to 10 nM TCDD by eliciting significant 400 and 40-fold increases in *CYP1A1* and *CYP1B1*, respectively (Fig. 2A, B). Increased expression of *CYP1A1* by indole was shown to be dependent on AHR activation through competitive antagonism with AHR antagonist GNF351 (Fig. S4)^24^. Further analysis of *AHR* mRNA expression in response to indole exposure revealed that the observed induction of *CYP1A1* and *CYP1B1* could not be attributable to enhanced AHR expression (Fig. 2C). Similar analysis of *Cyp1a1* and *Cyp1b1* expression performed in the mouse Hepa1 cell line exposed to indole failed to recapitulate the induction of *CYP1A1/B1* observed with human Caco2 cells, further demonstrating species-specific AHR activation by indole (Fig. S5). Examination of human Caco2 CYP1A1 enzymatic activity using an EROD-based methodology demonstrated that indole-mediated *CYP1A1* induction is not restricted to mRNA but is reflected at the level of CYP1A1 protein/activity. In response to 12 h exposure to 100 μM indole, CYP1A1 enzymatic activity is significantly enhanced ~4-fold over vehicle treated controls (Fig. 2D). This indole-mediated induction proved to be lower than that obtained with 10 nM TCDD, which may be a function of the half-life of indole when compared to the poorly metabolized TCDD.

In addition to stimulating direct DRE-mediated transcription, activated AHR has recently been demonstrated to act in a combinatorial fashion with inflammatory cytokine signaling to facilitate synergistic induction of interleukin-6 (*IL6*)^25^. We therefore examined the capacity of indole to activate AHR-mediated gene expression within the more complex context of *IL6* synergy. Caco2 cells were exposed to vehicle, 10 ng/ml IL1B, 20 μM indole, or a combination of IL1B together with indole, as indicated, and followed by quantitative PCR analysis of *IL6 *mRNA (Fig. 2E). Exposure to indole failed to elicit a significant induction of *IL6*; however combinatorial treatment with indole and IL1B prompted a robust and significant 3-fold synergistic induction of *IL6* expression when compared to IL1B treatment alone. In order to demonstrate AHR-dependency with regard to synergistic IL6 expression by indole and IL1B, we utilized the AHR competitive antagonist GNF351^24^. Caco2 cells were pre-treated with 200 nM GNF351 for 1 h prior to 20 μM indole and 10 ng/ml IL1B exposure for an additional 4 h (Fig. 2E). Treatment with GNF351 significantly suppressed the indole/IL1B-mediated synergistic induction of *IL6* by 50% without influencing the stimulatory action of IL1B in isolation. This observation indicates that the indole component of *IL6* synergy is dependent upon human AHR.

To establish whether indole/IL1B-mediated *IL6* induction by Caco2 cells is reflected at the protein level, an IL6 ELISA assay was performed. Caco2 cells were treated with vehicle, 10 nM TCDD or 100 μM indole, in isolation or in combination with 10 ng/ml IL1B, as indicated (Fig. 2F). Following 24 h treatment, conditioned media was collected and assayed for secreted IL6 protein. Data obtained were consistent with AHR agonist-mediated synergistic induction of *IL6* mRNA. In the context of IL1B exposure, both TCDD and indole stimulated IL6 protein secretion ~3-fold over that observed with IL1B alone, indicating that IL6 protein synthesis is influenced by indole exposure.

### Primary macrophages expressing the human AHR are permissive to indole induced receptor activity

To examine human AHR activation by indole in non-transformed cell lines, we utilized primary peritoneal macrophages (Mϕ) derived from commercially available ‘humanized’ AHR mice compared with wild-type C57BL6 mice expressing the mouse AHR. Expression of human and mouse AHR by ‘humanized’ AHR and control mice, respectively, was established through AHR-specific immunoblot analysis (Fig. S6). Peritoneal Mϕ derived from ‘humanized’ and wild-type controls were exposed (4 h) to vehicle, 10 μM indole or 500 pM indirubin and *Cyp1a1* mRNA expression quantified though PCR (Fig. 2G). Exposure to indirubin elicited a significant 20-fold increase in *Cyp1a1* expression by ‘humanized’ AHR Mϕ but limited ability to induce in wild-type Mϕ, consistent with previous reports demonstrating human AHR-selective activation by indirubin, thus demonstrating ‘humanized’ AHR Mϕ are fully permissive for AHR-mediated transcription by human-selective activators^26^. Exposure to indole stimulated *Cyp1a1* expression in ‘humanized’ AHR Mϕ by 5-fold but exhibited an attenuated ability to induce expression in identically treated wild-type Mϕ. These data further demonstrate that indole exhibits a capacity to activate AHR-mediated transcription in a species-selective manner and support that the hypothesis that indole would modulate immune cell activity through AHR activation in humans and to a lesser extent in rodents.

### Activation of human AHR-mediated transcription by indole is a consequence of direct ligand binding

**Figure 3:**
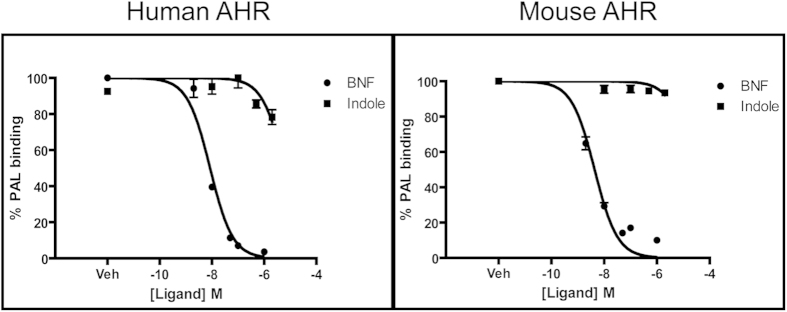
Indole is a human specific AHR ligand. Photoaffinity ligand binding competition assay in which increasing amounts of βNF and indole were added to hAHR or mAHR liver cytosol in combination with a fixed amount of the photoaffinity ligand to evaluate relative competition of indole within the ligand binding pocket of AHR between species. Higher concentrations of competing ligand were not tested as concentrations above 10 μM can yield non-specific competition.

Species-specific activation of human AHR-mediated transcription indicated that indole is a putative AHR ligand. To investigate this notion further, competitive ligand binding assays were performed utilizing hepatic cytosol derived from mice expressing the human *AHR* transgene under the control of the hepatocyte-specific *albumin* promoter (Fig. 3). Human AHR liver cytosol, incubated under saturating conditions with the AHR photoaffinity ligand (PAL) and increasing concentrations of indole, revealed a dose-dependent decrease in PAL binding consistent with indole being a competitor and direct ligand for the human AHR. A comparison of the binding affinities of indole with the known AHR ligand beta-napthoflavone (βNF) suggests that the relative affinity of human AHR for indole is orders of magnitude lower than for βNF. Competitive ligand binding assays performed using hepatic cytosol derived from wild-type C57BL6 mice demonstrated the expected competition with βNF, but showed a lack of indole-mediated competition at the doses examined. Higher concentrations of indole were not examined due to non-specific effects upon PAL binding. The absence of indole-mediated competition with wild-type murine AHR is consistent with previous reporter gene expression and quantitative PCR analysis, which demonstrate that indole is not an effective activator of the mouse AHR (Fig. 3).

**Figure 4:**
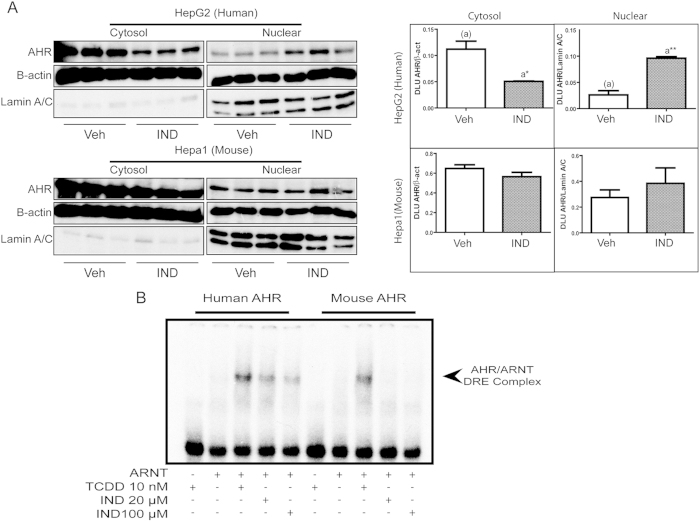
Indole facilitates human specific AHR nuclear localization and DRE binding capacity. (**A**) Nuclear translocation of AHR was determined following indicated treatment (1 h) in HepG2 (human) and Hepa1 (mouse) cell lines via western blot analysis. Relative quantification of AHR (normalized to β-actin or Lamin A/C) was determined via Phosphoimager and OptiQuant software, and presented as digitized light units (DLU). (**B**) *In vitro* translated hAHR/ARNT gel shift assay displaying treatment capacity to transform hAHR or mAHR to AHR/ARNT/DNA complex.

Additional evidence for the human AHR agonist potential of indole was obtained by performing nuclear translocation and DNA binding assays. Sub-cellular localization of AHR in human-derived HepG2 cells incubated (1 h) with vehicle or 100 μM indole was assessed by immunoblotting and demonstrated a redistribution of AHR from the cytoplasm into the nucleus upon treatment with indole, consistent with the action of an AHR agonist (Fig. 4A). Similar localization studies performed using mouse-derived Hepa1 cells failed to exhibit significant nuclear enrichment of AHR following incubation with indole (Fig. 4A). Consistent with the observed human-specific nuclear redistribution of AHR following exposure to indole, DNA retardation assays exhibited the capacity of indole to facilitate binding of *in vitro* translated human AHR/ARNT but not mouse AHR/ARNT to its cognate DNA response element (Fig. 4B).

### Indole derivatives exhibit structure-activity relationships with regard to human AHR activation

**Figure 5:**
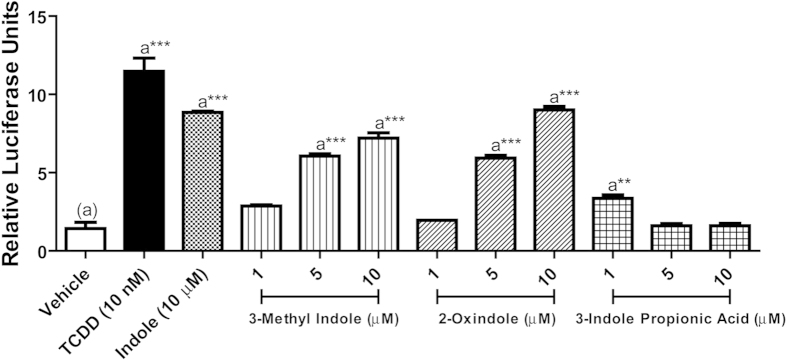
Ligand specificity of hAHR for microbiota-derived indoles. HepG2 (40/6) cells were treated as indicated for 4 h; cells were lysed, and luciferase activity was measured.

**Figure 6:**
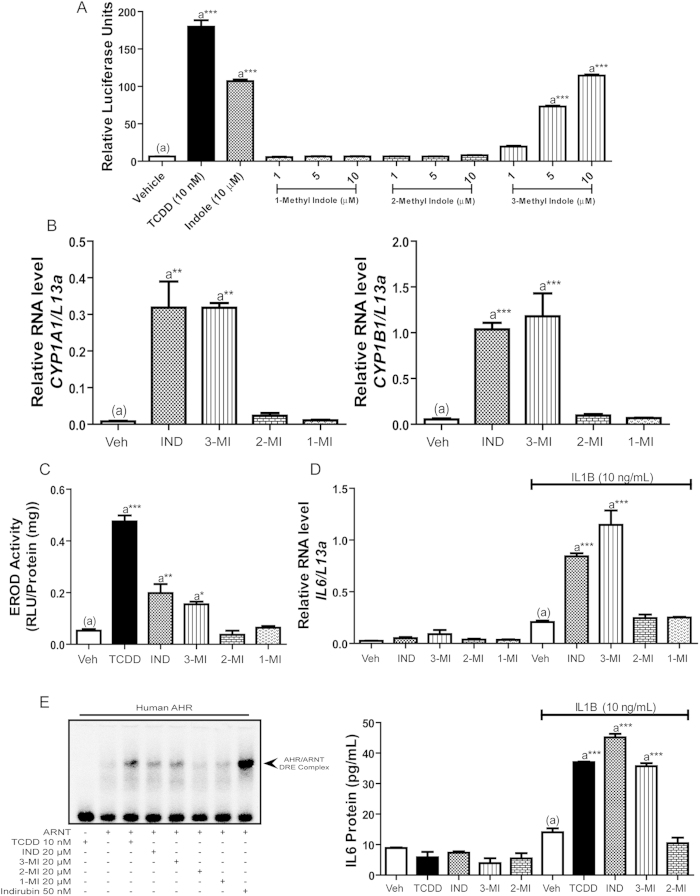
Methyl-indole isomers exhibit differential capacity to mediate AHR activity. (**A**) HepG2 (40/6) cells were treated as indicated for 4 h; cells were lysed, and luciferase activity was measured. (**B**) Expression of AHR-responsive *CYP1A1* and *CYP1B1* within Caco2 cells was determined through qPCR analysis following 4 h of treatment with vehicle, indole (IND), 3-methyl indole (3-MI), 2-methyl indole (2-MI), or 1-methyl indole (1-MI) at a concentration of 20 μM. (**C**) The mean CYP1A1 enzymatic activity was measured in Caco2 cells following 12 h treatment with DMSO, TCDD (10 nM), or indole/methyl indole isomers (100 μM) and 3 h incubation with luciferin-CEE reagent. (**D**) Synergistic *IL6* expression within Caco2 cells was determined by qPCR following 4 h treatment with vehicle, TCDD (10 nM), or indole/methyl indole isomers (20 μM) with or without the addition of IL1B (10 ng/mL). IL6 secretion by Caco2 cells was determined by ELISA following 24 h treatment with vehicle, TCDD (10 nM) or indole/ methyl indole isomers (100 μM) with or without the addition of IL1B (10 ng/mL). (**E**) *In vitro* translated AHR/ARNT gel shift assay displaying treatment capacity to transform human AHR to AHR/ARNT/DNA complex.

The establishment of indole as an agonist for the human AHR raised the question: are there other biologically relevant indole derivatives that are ligands for the human AHR? To address this question, HepG2 (40/6) cells were incubated with vehicle, 10 nM TCDD, 10 μM indole, 1–10 μM 3-methyl indole (skatole), 2-oxindole or 3-indole propionic acid, as indicated and luciferase reporter activity determined. The data obtained demonstrates that 3-methyl indole and 2-oxindole can stimulate the human AHR, with both indole derivatives exhibiting a dose-dependent increase in luciferase reporter activity and an efficacy equivalent to indole (Fig. 5). In contrast, 3-indole propionic acid stimulated reporter activity but failed to exhibit dose-dependency. Complementary studies performed using Hepa 1.1 cells largely failed to identify a stimulatory effect associated with the indole derivatives (Fig. S7). However, 3-methyl indole does exhibit modest dose-dependent activation of the mouse AHR. Structure-activity relationships of human AHR for indole derivatives were investigated further by exposure of human HepG2 (40/6) luciferase reporter cells to isomers of methyl indole (1-methyl indole, 2-methyl indole and 3-methyl indole). Cells were treated with vehicle, 10 nM TCDD, 10 μM indole or 1–10 μM methyl indole isomers, as indicated (Fig. 6A). The data reveal a dose-dependent increase in AHR activity associated with exposure to 3-methyl indole that is equivalent to that observed with indole. However, no significant AHR activity was evident with either 1-methyl or 2-methyl indole. Identical structure-activity associations were observed by analyzing *CYP1A1*/*B1* mRNA expression together with CYP1A1 enzyme activity (Fig. 6B,C) and synergistic *IL6* mRNA/protein expression in Caco2 cells (Fig. 6D). Electrophoretic mobility shift assays (EMSA) demonstrated that the elevated AHR activity observed with 3-methyl indole, like indole was associated with enhanced binding of AHR/ARNT to its cognate response element (Fig. 6E). Complementary studies performed using Hepa1 cells revealed no significant AHR activity following exposure to indole or any of the isomers of methyl indole tested (Fig. S7).

### *In silico* modeling predicts the structure-activity selectivity of indole and 3-methyl indole associated with human AHR

**Figure 7:**
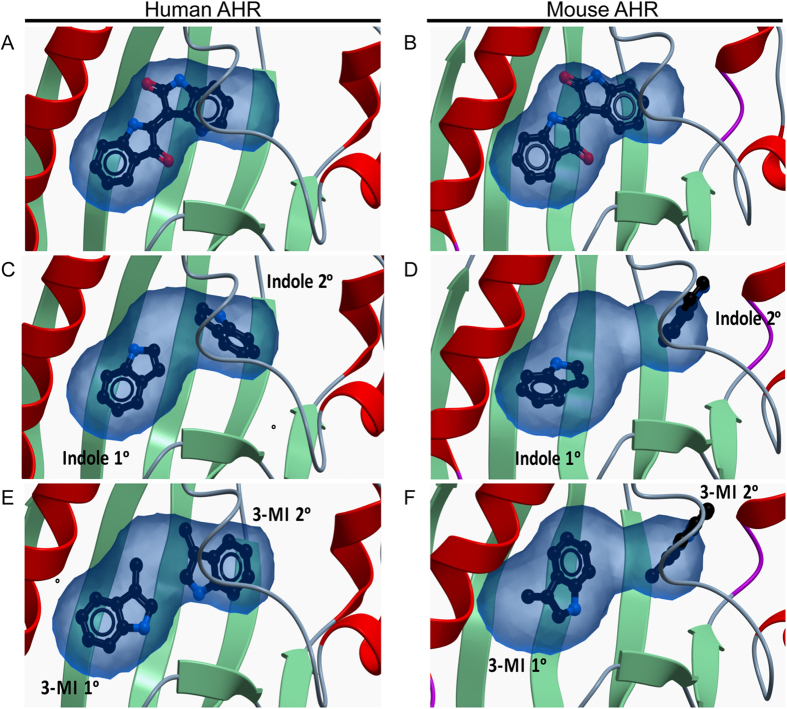
*In silico* modeling of AHR ligand binding domain. Homology modeling of indirubin optimized ligand binding in (**A**) hAHR and (**B**) mAHR. The predicted two indole-binding model in (**C**) hAHR and (**D**) mAHR ligand binding domain. The predicted two 3-methyl indole-binding models in (**E**) hAHR and (**F**) mAHR ligand binding domain. Blue shading indicates the space-filling volume of the ligand binding pocket.

The data suggest that the AHR activities associated with both indole and 3-methyl indole are selective for the human AHR. Furthermore, direct ligand binding and subsequent nuclear translocation and DNA binding facilitate the enhanced transcriptional activity of human AHR elicited by indole. In an effort to understand the molecular basis for such ligand binding and selectivity, *in silico* modeling of human and mouse AHR ligand binding domains (LBD) (amino acid residues 247–290 or 241–284, human or mouse respectively) was conducted in the context of indole and 3-methyl indole (Fig. 7). Homology modeling of human and mouse AHR LBD docked with indirubin provided an optimized model exhibiting the most energetically favorable LBD conformation for ligand binding (Fig. 7A,B). Subsequent docking simulations, using these optimized LBD conformations in the context of indole, 3-methyl, or 2-methyl indole binding, revealed no significant difference in LBD conformation or free energy calculations that can account for the observed *in vitro* experimental evidence indicating human AHR selectivity (Fig. S8). The previously established specificity of the human AHR for indirubin, which closely resembles two covalently linked indole moieties, suggested the novel concept that stoichiometry of human AHR/indole binding may be 2:1 rather than 1:1. This two indole binding hypothesis was examined using the *in silico* LBD models of human or mouse AHR and identified a favorable conformation associated with two molecules within the human but not the mouse AHR LBD (Fig. 7C,D; Table S1). Similar modeling predictions were performed using 3-methyl indole, which exhibits human AHR-selective activity *in vitro* (Fig. 7E). Data obtained substantiate the two indole binding hypothesis, a favorable conformation was observed for human AHR and 3-methyl indole with a simulated stoichiometry of 2:1. However, this stoichiometry was not permissive when modeled with the mouse AHR LBD (Fig. 7F). Binding of 2-methyl indole moieties at this ratio proved to be energetically unfavorable in the context of human AHR.

### Activation of AHR by indole is conserved across hominids

Activation of human AHR by indole but not mouse AHR represents a gain of function for the human AHR and indicates an evolutionary divergence within the *Ahr* locus. In order to examine whether this divergence is a specialization restricted to humans and therefore a recent adaptation, or a characteristic of the *Hominidae* family of primates in general and therefore a more distant mammalian divergence, we investigated the capacity of indole to activate *Pan troglodytes* (Chimpanzee) AHR. Treatment of *in vitro* translated mouse or chimpanzee AHR with vehicle, 10 nM TCDD or 20 μM indole, followed by EMSA, revealed chimpanzee AHR to be sensitive to both TCDD and indole-mediated activation, as evidenced by a robust level of AHR/ARNT/DRE complex formation (Fig. S9). *In silico* modeling of the chimpanzee AHR ligand binding domain in the context of indole identified the favorable bimolecular binding conformation previously observed with human AHR (Fig. S9). A comparison between the amino acid sequences that comprise the ligand binding domain of chimpanzee, human and mouse AHR reveals near 100% homology between chimpanzee and human, with only a single residue substitution at position 381 (valine and alanine, human, chimpanzee respectively) (Fig. S9). However, the homologous residue in mouse AHR is also an alanine residue and therefore is unlikely to be a determinant of indole binding.

## Discussion

The AHR, consistent with its established function as a xenobiotic sensor, exhibits a marked degree of promiscuity with regard to ligand binding^27^,^28^. Here we demonstrate that indole, an aromatic heterocyclic product of microbiota tryptophan metabolism, has the capacity to activate AHR-mediated transcription. Furthermore, the binding of indole and subsequent activation of AHR exhibits species dependency, with human AHR being permissive for activation while mouse AHR lacks the capacity to bind indole effectively. This finding is contrary to prevailing evidence which suggests that for many AHR ligands, the mouse AHR exhibits higher affinity than human AHR^29^. Notwithstanding, such species restriction is not without precedent; indirubin, a constituent of the *Indigofera* genus of plants and indoxyl-3-sulfate both exhibit higher activation potential for human AHR^13^,^26^. Initial *in silico* modeling failed to account for the species-specificity of indole binding, since models predicted similar binding for both AHR species. The low molecular weight of indole, compared to typical high affinity AHR ligands, combined with the relatively large ligand binding pocket of AHR, which is mostly conserved across species suggested that accommodation of indole would likely be conserved across mouse and human AHR. Thus, the confounding observation that indole theoretically binds both species of AHR yet selectively activates human AHR suggests a complex mode of selectivity rather than a simple binding or non-binding model for human and mouse, respectively. The capacity of the human AHR to accommodate indirubin combined with the structural similarity between indirubin and two indole moieties did not escape our attention^26^. This raised the intriguing notion that the molecular basis for indole selectivity may rely upon the binding of two molecules of indole within the ligand-binding pocket. Indeed, *in silico* modeling and docking provided supporting evidence indicating that the human AHR ligand binding pocket can adopt an energetically favorable conformation that is permissive for two molecules of indole, whereas the mouse AHR is more restrictive, allowing only a single indole to bind, resulting in very weak agonist activity. Importantly, further support of this bimolecular binding theory was obtained with mono-substituted methyl indole derivatives, whereby the modeling agreed with experimental evidence that demonstrated human-specific binding and AHR activation with 3-methyl indole but not 2-methyl or 1-methyl isomers. Such bimolecular ligand accommodation by the AHR ligand binding pocket has not previously been observed or considered and may greatly expand the opportunities for targeted modulation of AHR function. It will be interesting to perform gain of function studies with the mouse AHR through the generation of chimeric receptors and point mutants to determine the exact amino acid residues involved to the ability of the human AHR to bind indole.

Recent evidence has implicated the AHR in protection from pathogenic intestinal infection and inflammation together with the maintenance of homeostatic symbiosis between the host and their commensal microbiota^18^,^19^,^20^,^21^,^22^,^23^. However, the identification and source of ligands required to activate the AHR within the various cell types that comprise the intestine has not been fully determined. Until recently, it was presumed that AHR activation is mediated through ingestion of plant-derived dietary ligands such as polyphenolic flavonoids (*e.g.* quercetin), or glucobrassicin-derived gastric acid condensation products (*e.g.* indolo-[3,2*b*]-carbazole)^30^,^31^. Additionally, food combustion products (*i.e.* through cooking) such as benzo(*a*)pyrene are likely to contribute to dietary ligand exposure in humans^32^. Numerous reports have suggested that lack of intestinal homeostasis is a contributing factor to the pathology of many diseases, including inflammatory bowel disease, Crohn’s disease, ulcerative colitis, obesity, alcoholic and non-alcoholic fatty liver disease^33^,^34^,^35^. As such, reliance on exogenous diet-derived ligands to achieve a protective effect of AHR in intestinal homeostasis may prove restrictive when nutrition is limited or sporadic. However, additional endogenous and pseudo-endogenous sources of AHR agonists have been identified, which may allow for continuity of AHR activation and maintenance of intestinal homeostasis. For example, kynurenic acid and kyneurenine, products of tryptophan dioxygenase and tryptophan pyrolase (indoleamine 2,3-dioxygenase) metabolic pathways have been established as AHR agonists^12^,^16^,^36^. In addition, an increasing number of microbial-derived (pseudo-endogenous) agonists have been characterized or inferred; including, 1,4-dihydroxy-2-naphthoic acid generated by the probiotic bacterium *Propionibacterium freudenreichi*, tryptamine and indole-3-acetate, extracted from mouse cecal/fecal microbiota, together with products derived from *Lactobacillus bulgaricus* OLL1181^15^,^17^,^19^,^20^,^23^. Importantly, some of these AHR ligands have been shown to confer protection in models of colitis^18^,^19^,^20^,^21^,^23^. Additional examples of microbial AHR agonist production occur at other barrier tissues, indirubin and malassezin produced by the yeast *Malassezia* on the skin are both potent AHR activators, although their influence upon skin biology has not been fully investigated^37^,^38^. Largely, the characterization of such microbial AHR ligands as beneficial for intestinal function was determined using mouse models, suggesting that mouse AHR is permissive for activation by these indolyl compounds. Indeed, indole has been demonstrated to influence epithelial barrier function in mice, which appears to be contrary to our findings that indole modestly activated the mouse AHR yet exhibits relatively potent binding and activation of the human AHR^11^. However, the mouse AHR can be modestly activated by indole and 3-methyl indole. Such observations would indicate that in mice, the biological activity of indole is likely mediated modestly by the AHR as well as through additional mechanisms. The identification of indole as a selective human AHR agonist therefore raises the question as to the nature of the selective pressure that prompted the evolutionary adaptation of human AHR to function more efficiently as an indole sensor.

The evolutionary conservation of AHR across species from invertebrates and vertebrates implies important biological functions associated with the AHR. However, the broad range of species sensitivity to various naturally occurring AHR ligands, including indole, suggests a degree of evolutionary adaptation by the AHR. Such adaptation of the human AHR and other hominid species (*e.g. P. troglodytes*) to bind the microbial tryptophan metabolites indole and 3-methyl indole, both abundantly generated within the gastrointestinal tract, may provide a foundation for the establishment of an axis to regulate intestinal physiology, which may confer an evolutionary advantage that is redundant in rodents. The nature of the advantage is speculative but may involve the microbiota-indole-AHR-mediated maintenance of intestinal homeostasis throughout a longer lifespan and greater exposure to intestinal insults or conversely that the longevity (and enhanced reproductive potential) associated with hominids, including humans, is dependent upon intestinal integrity. Indeed, evidence linking intestinal homeostasis and longevity has been observed with *Drosophila*^39^,^40^.

In summary, we highlight the adaptation of the human AHR to bind and function as an indole receptor through a unique bimolecular mechanism to facilitate AHR-dependent gene expression, thus adding indole to the increasing compendium of ligands that can modulate human AHR activity. Given the abundance of indole-generating enteric bacteria and the high concentration of indole within the human intestinal tract, it is likely that indole stimulates AHR-dependent signaling. Future studies utilizing ‘humanized’ AHR mice will likely demonstrate that indole potentiates intestinal immunity, barrier integrity and overall intestinal health in a human AHR-dependent fashion.

## Methods

### Animals

C57BL/6J, *AHR*^*Ttr*^*Ahr*^*fx/fx*^*Cre*^*Alb*^, and Taconic^©^ C57BL/6-*Ahrtm1.1(AHR)Arte* mice were housed on corncob bedding in a temperature- and light-controlled facility and given access to food and water *ad libitum*. Mice were maintained in a pathogen-free facility and treated humanely with approval from the Animal Care and Use Committee of the Pennsylvania State University and methods were carried out in accordance with approved guidelines. Adult (10–12 weeks) mice were used for macrophage isolation experiments.

### Cell Culture

Hepa1, HepG2 and their respective AHR-reporter derivatives harboring the stably integrated pGudluc 1.1 or 6.1 constructs were maintained in α-modified essential media (Sigma-Aldrich, St. Louis, MO) supplemented with 8% fetal bovine serum (Hyclone Laboratories, Logan,). The Caco-2 human colon carcinoma cell line was maintained in α-MEM with 20% FBS. Primary peritoneal Mϕ cells were maintained in DMEM (Gibco, Carlsbad, CA) supplemented with 10% fetal bovine serum (FBS) (Hyclone Labs, Logan, UT), 2 mM L-Glutamine, and 1 mM sodium pyruvate (Sigma, St. Louis, MO). Cells were cultured at 37 °C in a humidified atmosphere composed of 95% air and 5% CO_2_ in the presence of 100 IU/ml penicillin/100 μg/ml streptomycin (Sigma-Aldrich).

### Primary peritoneal macrophage isolation from mice

Mice (m*Ahr*^*b*^ and h*Ahr*) were injected with 3 ml of 3% thioglycolate media intraperitoneally on day one. Approximately 72 h post-thioglycolate injection, mice were euthanized. Primary Mϕ were isolated by peritoneal lavage in ice-cold phosphate buffered saline (PBS). Cells were centrifuged and re-suspended in Mϕ culture media for 4 h. After 4 h cells were washed with PBS and incubated overnight in Mϕ media^41^. Cells were treated the following day for 4 h unless otherwise described in figure legends.

### PAL Ligand Competition assay

Characterization of competitive binding within the AHR ligand binding pocket between the AHR photoaffinity ligand, 2-azido-3-[^125^I]iodo-7,8-dibromodibenzo-*p*-dioxin and indole was performed essentially as described previously^26^.

### Luciferase Reporter Assays

The reporter cells (Hepa 1.1/Hep G2 40/6) were seeded in twelve-well plates and cultured to 90% confluence. Cells were treated as indicated for 4 h then lysed in 400 μl of lysis buffer [25 mM Tris-phosphate, pH 7.8, 2 mM dithiothreitol, 2 mM 1,2-diaminocyclohexane-*N*,*N*,*N*′,*N*′-tetraacetic acid, 10% (v/v) glycerol, and 1% (v/v) Triton X-100]. Lysate (20 μl) was combined with 80 μl of Luciferase Reporter Substrate (Promega, Madison, WI), and luciferase activity was measured with a TD-20e luminometer (Turner Designs, Sunnyvale, CA).

### Quantitative PCR Analysis

Total RNA was isolated from cells using TRI Reagent (Sigma-Aldrich), followed by reverse transcription using the High Capacity cDNA Archive kit (Applied Biosystems, Foster City, CA) according to the manufacturer’s protocols. PerfeCTa SYBR Green SuperMix for iQ (Quanta Biosciences, Gaithersburg, MD) was used to determine mRNA levels, and analysis was conducted using MyIQ software, in conjunction with a MyIQ-single-color PCR detection system (Bio-Rad Laboratories, Hercules, CA).

### ELISA

To quantify protein expression, media was collected from Caco-2 cells at 12 and 24 h post treatment and stored at −80 °C. IL6 content of media samples was determined via LEGEND MAX™ Human IL6 ELISA kit with pre-coated plates (Biolegend, San Diego, Ca.) according to manufacturer’s protocol.

### CYP1A1 Activity Assay

Activity of CYP1A1 was determined using P450-Glo™ CYP1A1 Assay following the manufacturer’s intstructions (Promega, Madison, WI). Caco-2 cells were cultured to 90% confluence, then treated with ligand as indicated for 12 h, followed by the addition of 5 μL (1:100 v/v) of Luc-CEE for an additional 3 h. This was followed by the addition of 150 μL of lysis buffer, chemiluminescence was determined by mixing 50 μL of lysate plus 50 μL of luciferase reporter substrate and measured on a TD-20e luminometer. The luciferase activity was normalized to protein content of the lysate as determined by BCA protein assay (Thermo Fisher Scientific, Waltham, MA).

### AHR Nuclear Translocation Analysis

HepG2 and Hepa1 cells were treated with vehicle or indole (100 μM) followed by nuclear/cytosol protein isolation and protein blot analysis, as described previously^42^.

### Electrophoretic Mobility Shift Assay

Gel retardation assays were performed using *in vitro* translated human AHR, mouse AHR and ARNT, as described previously^43^.

### In Silico Modeling

The homology model of mouse and human AhR-PASB-LBD based on the NMR *apo* of the HIF-2α-PASB (PDB 1P97) was prepared and optimized as recently described^44^. Indirubin was docked into the mouse and human optimized model. Then, the complexes were submitted to 1.5 × 10^4^ steps MC ligand-protein side chain optimization to reach the most energetically favorable conformations. Molecular docking was run as previously reported^45^,^46^,^47^,^48^. In the ICM-VLS (Molsoft ICM) screening procedure, the ligand scoring is optimized to obtain maximal separation between the binders and non-binders. Each compound is assigned a score according to its fit within the receptor; this ICM score accounts for continuum and discreet electrostatics, hydrophobicity and entropy parameter. Surface energy of binding is based on atomic solvent-accessible surface using special water molecule probe radii designed for calculations of the solvation energy. This term based on “*atomic solvation*” is a product of atomic accessibilities by the atomic energy density parameters similar to those proposed in literature^49^. The non-hydrogen atomic accessible surfaces are calculated using a faster modification of the Shrake & Rupley-algorithm^50^. The energy value calculated is the difference between the two molecules and the sum of the one molecule-free state (ΔH, kcal/mol).

### Data Analysis

Data analysis was conducted using Prism 4 software, GraphPad Software Inc. (San Diego, CA). One Way ANOVA analysis was completed using Bonferroni post test. T-test parameters were unpaired and two-tailed analysis. Data represent mean ± S.E.M. and are representative of three independent experiments *p*-value ≤0.05 (*), *p*-value ≤0.01 (**), *p*-value ≤0.001 (***).

## Additional Information

**How to cite this article**: Hubbard, T. D. *et al.* Adaptation of the human aryl hydrocarbon receptor to sense microbiota-derived indoles. *Sci. Rep.* **5**, 12689; doi: 10.1038/srep12689 (2015).

## Supplementary Material

Supplementary Information
