# Supplementary material for: Adaptation of the human aryl hydrocarbon receptor to sense microbiota-derived indoles
Source: Sci Rep. 2015 Aug 3;5:12689. doi: 10.1038/srep12689 (PMC4522678; doi:10.1038/srep12689)
Supplement: Supplementary Information [file srep12689-s1.pdf]

SUPPLEMENTARY MATERIALS AND METHODS, TABLE, FIGURES AND LEGENDS

'Adaptation of the human aryl hydrocarbon receptor to sense microbiota-derived indoles'

Troy D. Hubbard, Iain A. Murray, William H. Bisson, Tejas S. Lahoti, Krishne Gowda, Shantu G. Amin, Andrew D. Patterson and Gary H. Perdew

MATERIALS AND METHODS

Indole Recrystallization/Purification: Commercial indole was purified by recrystallization using chloroform/petroleum ether and the pure compound was characterized by ^{1}H and ^{13}C NMR. ^{1}H NMR (500 MHz, $CDCl_3$) δ 6.62 (s, 1H), 7.14-7.17 (t, 1H, J = 7.5 Hz), 7.20-7.26 (m, 2H), 7.40 (d, 1H, J = 8 Hz), 7.68 (d, 1H, J = 7.5 Hz), 8.09 (s, 1H); ^{13}C NMR (500 MHz, $CDCl_3$) δ 102.66, 111.04, 119.85, 120.75, 122.02, 124.13, 127.89, 135.81; mp = 52-54 ºC. Further, the purity was confirmed by reverse-phase HPLC using 4.6 mm × 25 cm vydac-C18 column eluted with a gradient from 30 to 100% CH_3OH in 30 min, the flow rate was 1 mL/min, and monitored at 254 nm, RT = 14.65 min. The purity level was > 99%.

Electrophoretic Mobility Shift Assay: Gel retardation assays were performed using *in vitro* translated human AHR, mouse AHR and ARNT protein generated via TnT® Coupled Reticulocyte Lysate Systems according to manufacturer's protocol (Promega, Madison, WI). Human AHR *in vitro* translations were supplemented with sodium molybdate to a final concentration of 1.25 mM to enhance stability of the receptor/HSP90 complex. Four microliters each of AHR and ARNT proteins were combined with 1.5 µL of HEDG buffer (25 mM HEPES, 1 mM EDTA, 10 mM sodium molybdate, and 10% glycerol), with the addition of indicated treatments (0.5 µL) and incubated at room temperature for 15 min. Following treatment incubation, 2x10^6 cpm of ^{32}P-DRE oligonucleotides were added to each reaction and incubated at room temperature for an additional 15 min. After addition of 2 µL of 0.25% xylene cyanol in 20% (w/v) Ficoll to the reaction mixture, samples were loaded onto a 6% non-denaturing DNA retardation gel (Invitrogen, Carlsbad, CA) and separated by electrophoresis. The gels were fixed in a 7:1:1:1 water:methanol:acetic acid:glycerol solution for 20 min, dried for 60 min, and then subjected to autoradiography for analysis.

Chimpanzee AHR cDNA synthesis: The codon-optimized chimpanzee AHR cDNA was synthesized by GenScript (Piscataway, NJ) and cloned into the HindIII and XhoI sites of pcDNA3, a mammalian expression vector.

Real time PCR Primers

<u>Human</u>

CYP1A1-F-5'-TAC CTC AGC CAC CTC CAA GAT-3'
CYP1A1-R-5'-GAG GTC TTG AGG CCC TGA TT-3'
CYP1B1-F-5'-TGC CTG TCA CTA TTC CTC ATG CCA -3'
CYP1B1-R-5'-ATC AAA GTT CTC CGG GTT AGG CCA -3'
AHR-F-5'-GAA GAT GGT GAT GGG ATT TC-3'
AHR-R-5'-GAA GGT GAA GGT CGG AGT -3'
IL6-F-5'-AAA TTC GGT ACA TCC TCG ACG-3'
IL6-R-5'-AGT GCC TCT TTG CTG CTT TCA -3'
L13A-F-5'-CCT GGA GGA GAA GAG GAA AGA GA-3'
L13A-R-5'-GAG GAC CTC TGT GTA TTT GTC AA -3'

<u>Mouse</u>

Cyp1a1-F-5'-CTC TTC CCT GGA TGC CTT CAA-3'
Cyp1a1-R-5'-GGA TGT GGC CCT TCT CAA ATG -3'
Cyp1b1-F-5'- GCT AGC CAG CAG TGT GAT GAT ATT-3'
Cyp1b1-R-5'-GGT TAG CCT TGA AAT TGC ACT GAT -3'
Ahr-F-5'-GCG TCA GCT ACC TGA GGG CCA-3'
Ahr-R-5'-GGG CCA TGG GCT TCG TCC AC-3'
L13a-F-5'-TTC GGC TGA AGC CTA CCA GAA AGT-3'
L13a-R-5'-GCA TCT TGG CCT TTT TCC GTT-3'

<u>m*Ccl20*_3.1kb DRE Oligo EMSA Sequence</u>

Ccl20-3.1kb DRE-F-5'-TTG TGT GTG TGC GTG TGT GCG TGT GTT AC-3'
Ccl20-3.1kb DRE-R-5'-TGT AAC ACA CGC ACA CAC GCA CAC ACA C-3'

Supplementary Table 1. Surface energy of binding (ΔH, kcal/mol) for 3MI and IND molecules into the mouse and human AHR PASB after second round of docking.

	**Mouse**	**Human**
**Indole**	-0.09	-0.58
**3MI**	-0.37	-1.76

Supplementary Figures

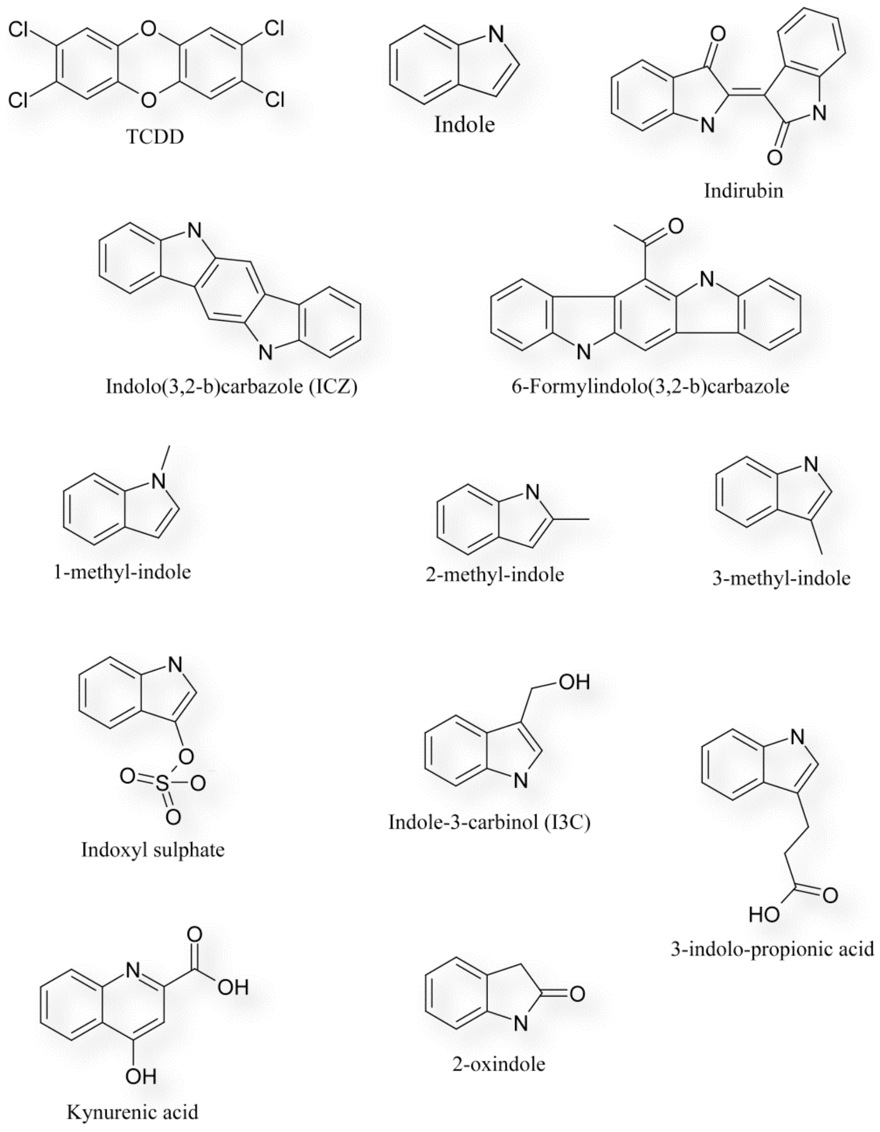

Figure S1: Molecular structure of AHR Ligands and Indoles.

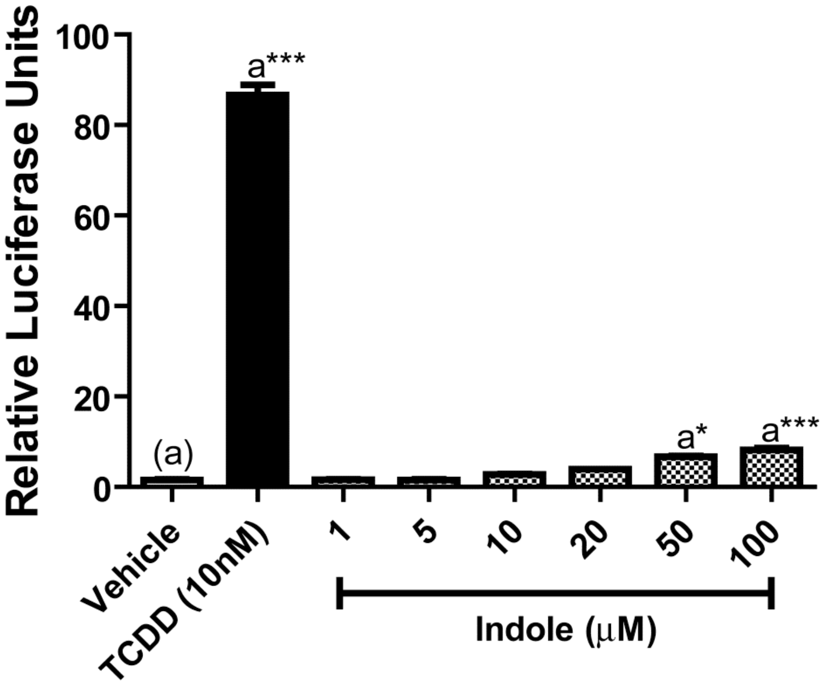

Figure S2: Indole marginally activates the AHR in a rat cell line. Indole dose-response assessment for AHR-dependent transcriptional activity in H4-II-E1.1(Rat) reporter cell line. The cells were treated as indicated for 4 h; followed by lysis and measurement of luciferase activity.

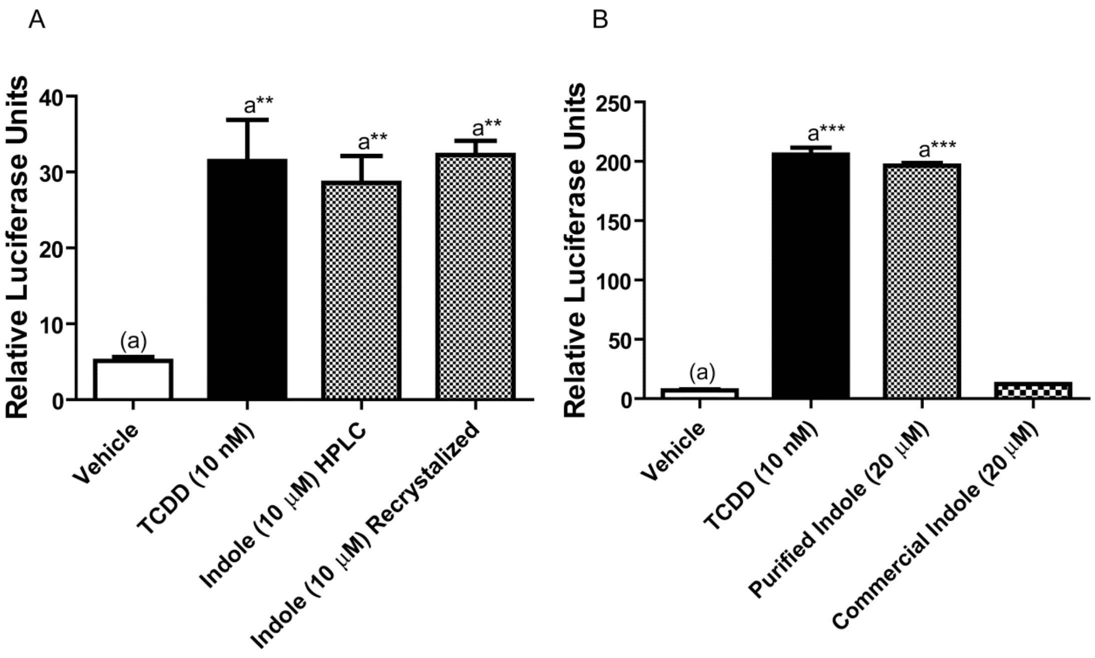

Figure S3: Purified indole induces AHR transcriptional activity. (A) Comparison of AHR dependent luciferase activity of recrystalized and HPLC pure indole and (B) purified indole compared with commercial indole within HepG2 (40/6) cells. Cells were treated as indicated for 4 h; followed by lysis and measurement of luciferase activity.

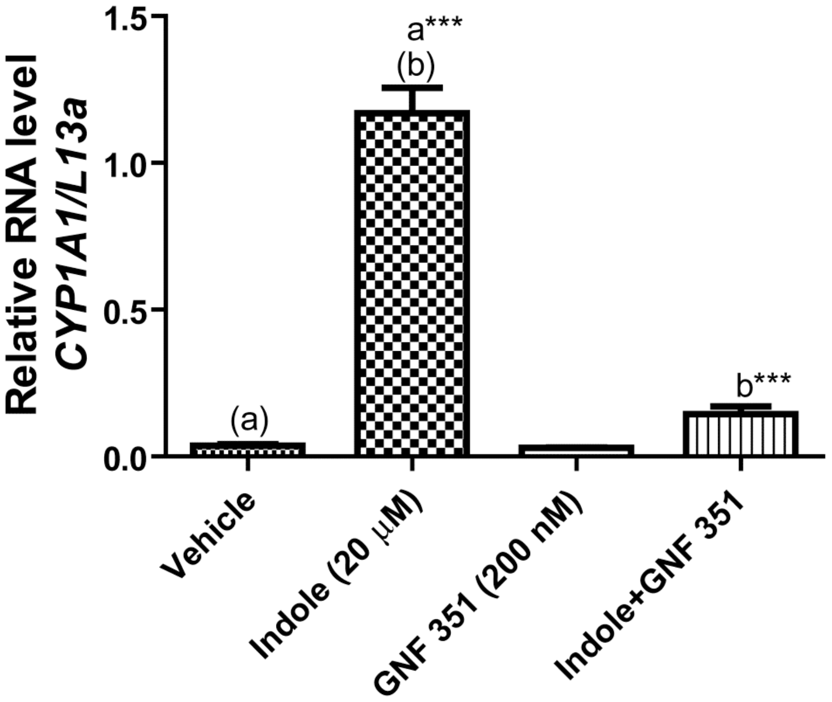

Figure S4: AHR antagonist GNF351 suppresses indole mediated induction of *CYP1A1* mRNA. Expression of AHR target gene *CYP1A1* within Caco2 cells was determined through qPCR analysis following 4 h of treatment with DMSO (Vehicle), TCDD (10 nM), or indole (IND) at the indicated dose with or without 1 h pretreatment with known AHR antagonist GNF351 (200 nM).

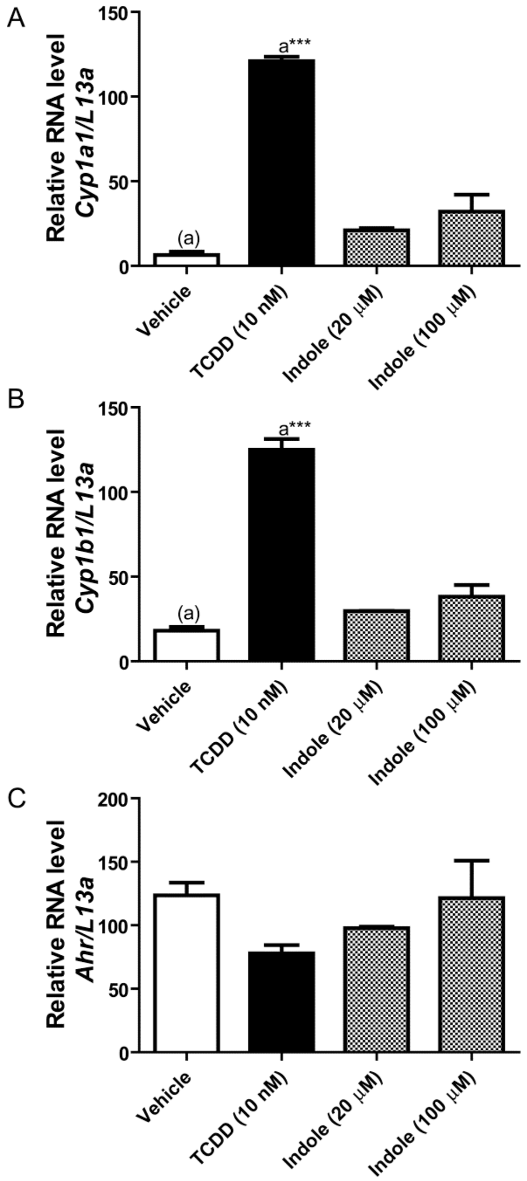

Figure S5: Indole fails to induce AHR target genes in mouse Hepa 1 cells. Expression of AHR-responsive (A) *Cyp1a1*, (B) *Cyp1b1*, and (C) *Ahr* within Hepa 1 cells was determined through qPCR analysis following 4 h of treatment with DMSO (Vehicle), TCDD (10 nM), or indole at the indicated dose.

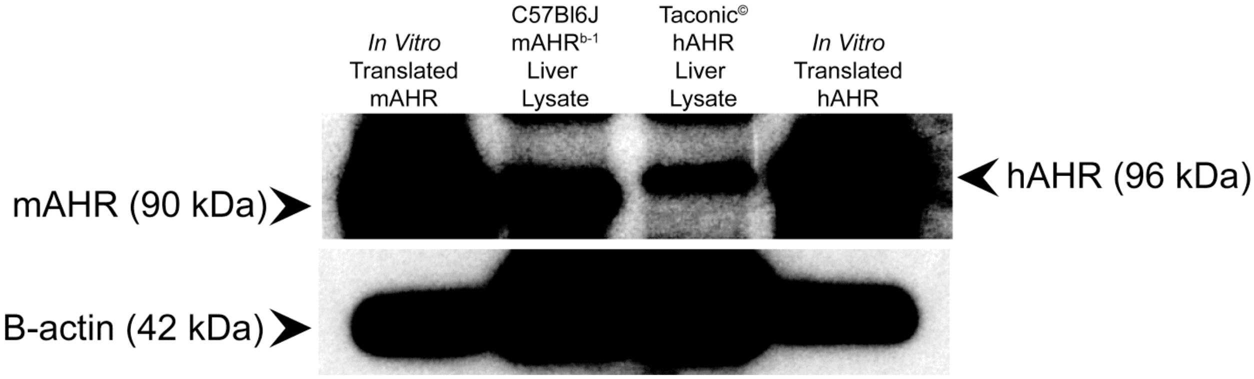

Figure S6: Western blot analysis of hAHR vs mAHR liver expression in C57BL/6J versus Taconic© AHR humanized mouse. Positive controls for human and mouse AHR were generated through *in vitro* translations.

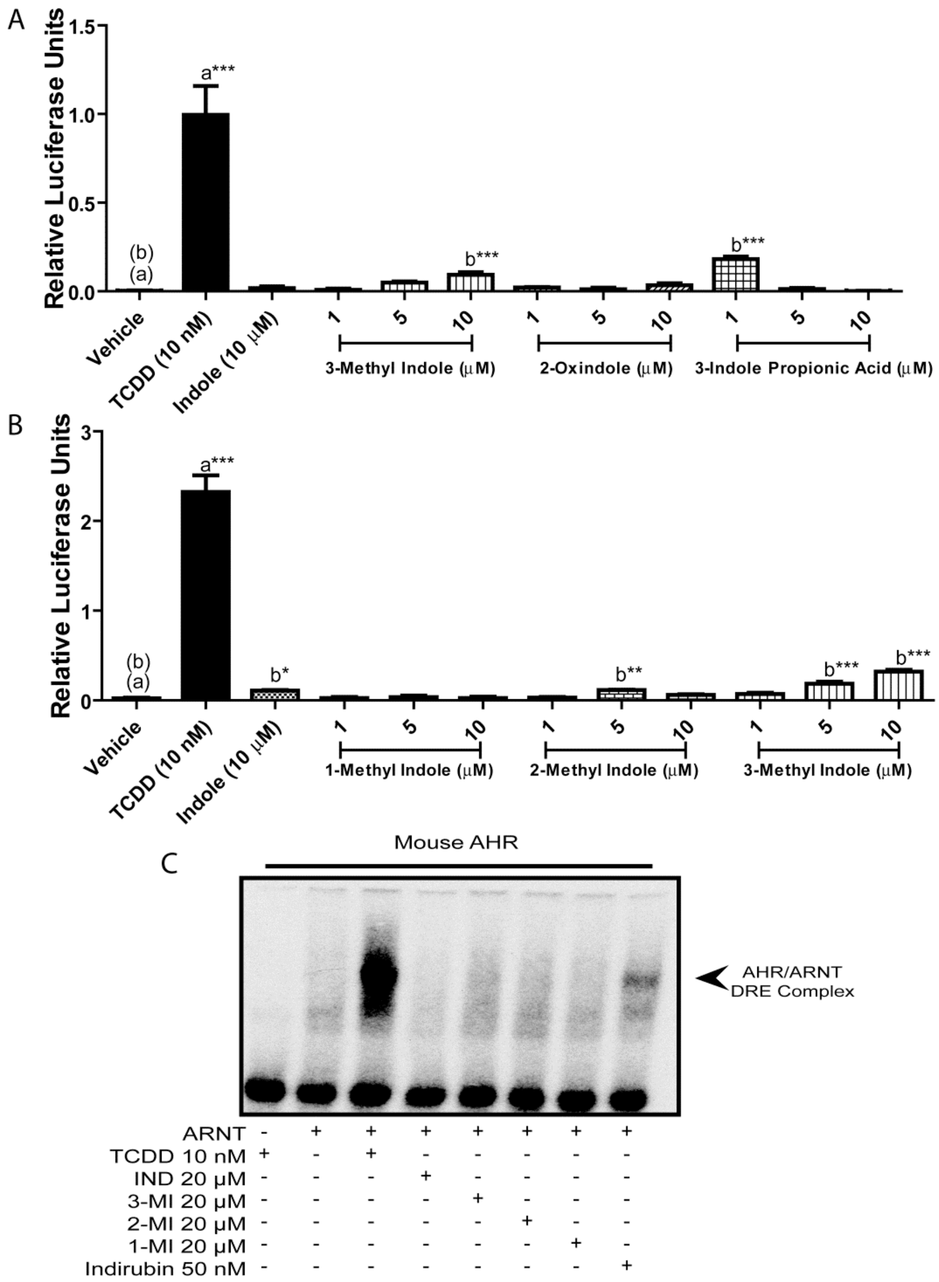

Figure S7: Mono-substituted indoles are poor inducers of mouse AHR transcriptional activity. (A) Hepa 1.1 reporter cells were treated as indicated for 4 h with the indicated microbial indole and (B) methyl indole derivatives, cells were lysed, and luciferase activity was measured. (C) *In vitro* translated mAHR/ARNT gel shift assay displaying treatment capacity to transform mouse AHR to AHR/ARNT/DNA complex. Indole (IND), 3-methyl indole (3-MI), 2-methyl indole (2-MI), 1-methyl indole (1-MI) and indirubin (IR).

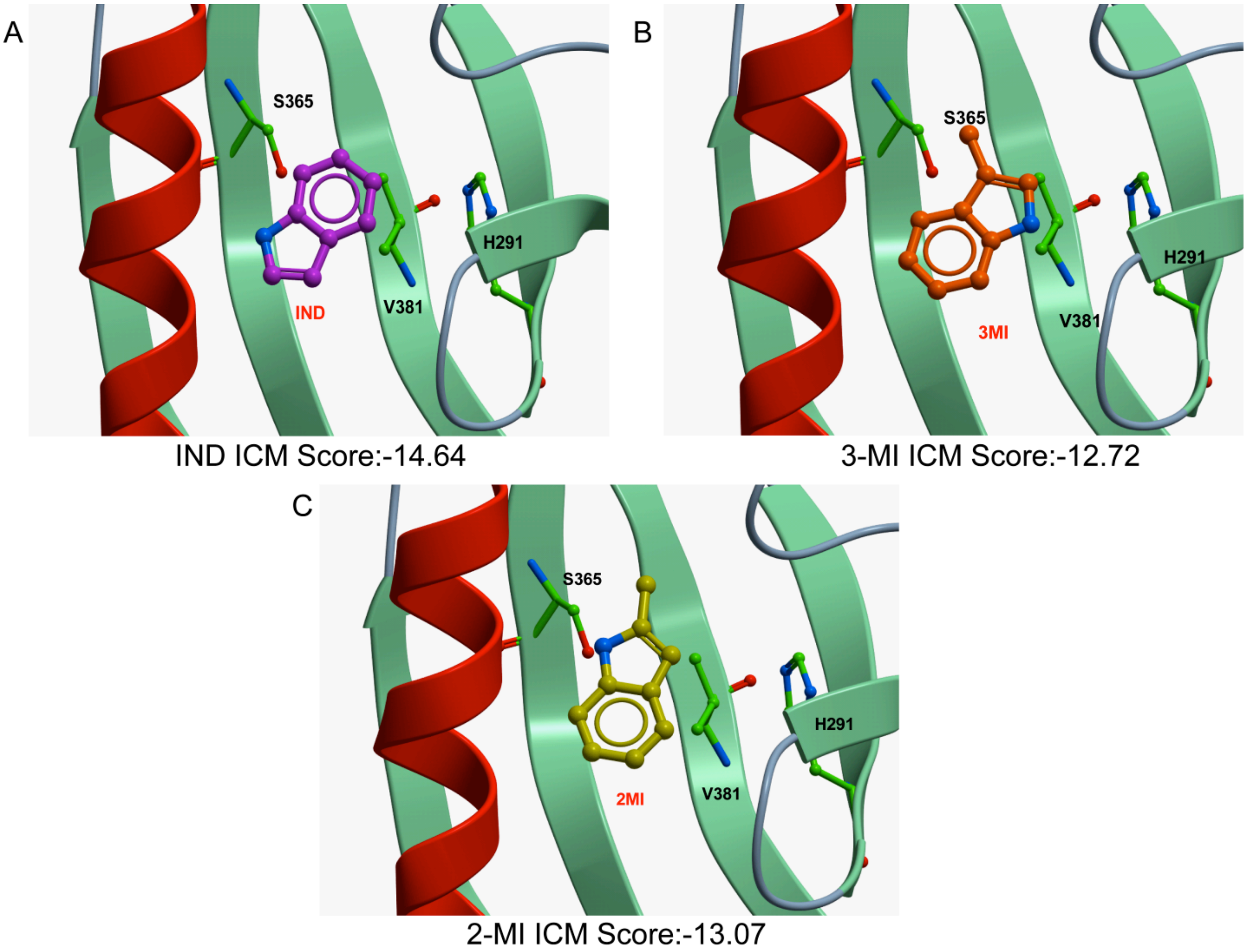

Figure S8: *In silico* modeling of a single indole molecule binding within hAHR ligand binding pocket. In silico modeling of (A) indole, (B) 3-methyl indole, or (C) 2-methyl indole binding within the human AHR ligand binding domain.

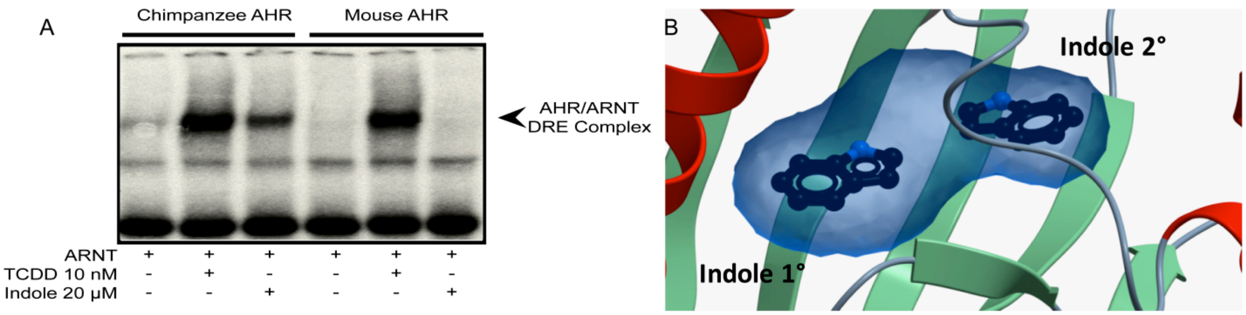

C

```
Chimpanzee  247-HGQKKKGKDGSILPPQLALFAIATPLQPPSILEIRTKNFIFRTKHKLDF-295
     Human  247-HGQKKKGKDGSILPPQLALFAIATPLQPPSILEIRTKNFIFRTKHKLDF-295
     Mouse  241-HGQNKKGKDGALLPPQLALFAIATPLQPPSILEIRTKNFIFRTKHKLDF-289
                   *      **

Chimpanzee  296-TPIGCDAKGRIVLGYTEAELCTRGSGYQFIHAADMLYCAESHIRMIKT-343
     Human  296-TPIGCDAKGRIVLGYTEAELCTRGSGYQFIHAADMLYCAESHIRMIKT-343
     Mouse  290-TPIGCDAKGQLILGYTEVELCTRGSGYQFIHAADILHCAESHIRMIKT-337
                         ***     *                * *

                                                     *
Chimpanzee  344-GESGMIVFRLLTKNNRWTWVQSNARLLYKNGRPDYIIATQRPLTDEE-390
     Human  344-GESGMIVFRLLTKNNRWTWVQSNARLLYKNGRPDYIIVTQRPLTDEE-390
     Mouse  338-GESGMTVFRLLAKHSRWRWVQSNARLIYRNGRPDYIIATQRPLTDEE-384
                     *     * **  *        * *        *
```

Figure S9: The chimpanzee AHR binds to and is activated by indole. (A) *In vitro* translated AHR/ARNT gel shift assay displaying indole capacity to transform chimpanzee AHR to AHR/ARNT/DNA complex compared to murine AHR. (B) *In silico* modeling of favorable binding of two indole molecules within the chimpanzee AHR ligand-binding domain. (C) Amino acid sequence comparison between the chimpanzee, human, and mouse AHR ligand binding domain.
